# On the modulation and maintenance of hibernation in captive dwarf lemurs

**DOI:** 10.1038/s41598-021-84727-3

**Published:** 2021-03-11

**Authors:** Marina B. Blanco, Lydia K. Greene, Robert Schopler, Cathy V. Williams, Danielle Lynch, Jenna Browning, Kay Welser, Melanie Simmons, Peter H. Klopfer, Erin E. Ehmke

**Affiliations:** 1 Duke Lemur Center, Durham, NC 27705 USA; 2 Department of Biology, Duke University, Durham, NC 27708 USA

**Keywords:** Physiology, Metabolism

## Abstract

In nature, photoperiod signals environmental seasonality and is a strong selective “zeitgeber” that synchronizes biological rhythms. For animals facing seasonal environmental challenges and energetic bottlenecks, daily torpor and hibernation are two metabolic strategies that can save energy. In the wild, the dwarf lemurs of Madagascar are obligate hibernators, hibernating between 3 and 7 months a year. In captivity, however, dwarf lemurs generally express torpor for periods far shorter than the hibernation season in Madagascar. We investigated whether fat-tailed dwarf lemurs (*Cheirogaleus medius*) housed at the Duke Lemur Center (DLC) could hibernate, by subjecting 8 individuals to husbandry conditions more in accord with those in Madagascar, including alternating photoperiods, low ambient temperatures, and food restriction. All dwarf lemurs displayed daily and multiday torpor bouts, including bouts lasting ~ 11 days. Ambient temperature was the greatest predictor of torpor bout duration, and food ingestion and night length also played a role. Unlike their wild counterparts, who rarely leave their hibernacula and do not feed during hibernation, DLC dwarf lemurs sporadically moved and ate. While demonstrating that captive dwarf lemurs are physiologically capable of hibernation, we argue that facilitating their hibernation serves both husbandry and research goals: first, it enables lemurs to express the biphasic phenotypes (fattening and fat depletion) that are characteristic of their wild conspecifics; second, by “renaturalizing” dwarf lemurs in captivity, they will emerge a better model for understanding both metabolic extremes in primates generally and metabolic disorders in humans specifically.

## Introduction

Seasonal environmental variation can impose energetic challenges on animals at predictable times of the year. Thus, it is not surprising that animals evolved mechanisms to tune reproduction, metabolic strategies, and other life-history traits to photoperiod^1^. However, in the absence of a changing photoperiod and the co-varying environmental conditions, e.g., under captive conditions, seasonal physiological responses expressed by animals vary depending on whether circannual clocks are endogenous or responsive to environmental input^2^.

Daily torpor and hibernation, two metabolic strategies employed by animals that face periodic environmental challenges such as food shortages, show physiologically distinctive patterns and are potentially regulated by different mechanisms^3–5^. Daily torpor generally involves shallow metabolic depression (e.g., high differential between body temperature and ambient temperature) that lasts for a few hours per day. Thus, daily torpor users can continue to forage during the active period of the day, while saving energy during the daily resting phase^6^. Daily torpor expression can occur throughout the year and irrespective of ambient temperature^5^. Daily torpor users may show evidence of fat deposits to fuel metabolism, but this is not a precondition for torpor expression, and some species enter torpor opportunistically without showing any evidence of fattening (e.g., Berthe’s mouse lemurs^7^). A more extreme example of metabolic depression is displayed by animals expressing multiday torpor, i.e., hibernation, which depress metabolism for weeks at a time. Hibernation is often seasonal, restricted to the wintertime, and is characterized both by deep metabolic depression and body temperatures that approximate ambient temperature^3,5^. Torpor bouts during hibernation are interspersed by periods of energetically expensive arousals^5^. Hibernators require prior accumulation of fat deposits to sustain months-long periods without feeding (unless they use food caches). Hibernators can, however, show short torpor bouts (less than 24 h) at the beginning and end of the hibernation season, or at high ambient temperatures, which may give the appearance of daily torpor^4^. As more studies of “non-holartic”^8^ heterotherms are accrued, however, it is becoming evident that daily torpor users and hibernators do not always fall into clear-cut categories^9–11^. In fact, there is growing evidence of heterotherms from all major phylogenetic lineages displaying extraordinary physiological flexibility: Marsupials like honey possums (Diprotodontia) can express daily torpor while significantly reducing body temperature to near ambient^12^; pygmy possums (Diprotodontia) can facultatively hibernate in the wild^13^; and hedgehogs (Eulipotyphla)^14^ and elephant shrews (Afrotheria)^15^ use daily torpor but can sporadically display prolonged torpor. One of the most impressive examples of heterothermic flexibility is shown by the common tenrec of Madagascar (Afrotheria) that can hibernate at an unprecedented range of body temperatures (from 12 to 28 °C) without undergoing sporadic arousals^16,17^.

Extreme heterothermic variation is also exemplified by Madagascar’s mouse lemurs (40–80 g), with members of the same species, e.g., *Microcebus griseorufus*, undergoing daily torpor, prolonged torpor, or hibernation, depending on their fat storage and environmental conditions^9^. The dwarf lemurs (*Cheirogaleus* spp*.*), phylogenetically related to mouse lemurs, are considered to be the only primates that express hibernation obligatorily: all species of *Cheirogaleus* for which we have data (7 out of 9 species) hibernate in the wild, displaying hibernation bouts that can last for a few days up to several months depending on hibernacula conditions^18^. The hibernation season lasts, depending on the species and individuals, between 3 and 7 months a year^19–22^. Prior to hibernation, all dwarf lemurs show significant fattening primarily concentrated in their tails. Fat deposits are then used to fuel metabolism during the hibernation season^20,23,24^.

Though the case of dwarf lemurs in the wild is a clear-cut example of hibernation, captive dwarf lemurs fail to express hibernation. A pioneer study conducted at the Duke Lemur Center (DLC, previously known as the Duke University Primate Center) showed that three fat-tailed dwarf lemurs subjected to constant photoperiodic (LD12/12), temperature, and dietary conditions showed seasonal changes in body mass, but inconsistent activity patterns: between September and December, the lemurs were sometimes lethargic, sometimes active^25^. No evidence of multiday torpor was reported. Similarly, in Brunoy, France, two captive dwarf lemurs subjected to more naturalistic conditions of alternating photoperiod and temperature variation between 24 and 30 °C (“summer” season) and 17 and 30 °C (“winter” season) failed to show a hibernation phase^26^. Dwarf lemurs did reduce activity levels between July and August and experienced a period of fattening prior to the expected “winter” season. The caveat, however, was that ambient temperature settings did not allow the dwarf lemurs to further reduce body temperature or depress metabolism (but see Petter-Rousseaux’s pers. comm. cited in^26^ on mention of hibernation in dwarf lemurs subjected to 12 °C). Contemporary work by McCormick^27^ also failed to confirm metabolic depression consistent with hibernation when oxygen consumption was measured in three dwarf lemurs at the DLC exposed to alternating photoperiod, relatively constant temperature conditions (~ 23 °C), and food ad libitum. Finally, Foerg and Hoffman^28^ did not find evidence of hibernation in DLC dwarf lemurs, corroborating previous studies. Although dwarf lemurs showed lethargic behavior, including reduced activity, prior to the reproductive season (February–March), some level of activity persisted throughout the study. It is important to note that none of these studies experimented with food restriction, nor did they reduce ambient temperature below 20 °C.

More than 20 years have passed since these pioneer studies, and questions still remain as to whether or not dwarf lemurs under captive conditions can express consistent hibernation. Krystal et al.^29^ showed that at least one dwarf lemur at the DLC decreased metabolic rates consistent with hibernation, but the lemur’s individual torpor bouts did not last longer than 24 h. Recent observations showed that some dwarf lemurs avoided eating for multiple days during the winter season, while maintaining skin temperatures close to cooler ambient temperatures (PHK, RS, unpublished). This study sought to examine the role of temperature and food availability but was challenged by sporadic sampling. Nonetheless, the results suggested that while those factors likely influenced the depth and duration of torpor, they were not essential to its initiation. The data suggested seasonal torpor, leading to hibernation, was the result of an endogenous cycle. This is consistent with the fact that other non-primate hibernators undergo physiological changes in preparation for hibernation, such as fattening, in the absence of environmental cues, suggesting that genetic mechanisms are at play^30^.

Available evidence to date is insufficient to confirm whether or not dwarf lemurs under captive conditions can express hibernation comparable to that of their wild counterparts. We argue that a “lack of hibernation” by dwarf lemurs in captivity could be due either to environmental constraints, i.e., conditions that are not conducive to hibernation expression, or biological constraints, i.e., captive-bred dwarf lemurs may have lost their capacity to hibernate. In all previous studies, ambient temperatures were maintained at relatively constant levels around 25 °C (likely close to the thermoneutral zone for cheirogaleids^31^), which may not facilitate multiday torpor expression. Moreover, food availability may have prevented torpor expression, either directly (by increasing metabolism) and/or indirectly (by increasing disturbance). Alternatively, it is possible that captive breeding and exposure to artificial conditions may hinder the dwarf lemurs’ ability to express hibernation. DLC dwarf lemurs have been captive bred for a minimum of four generations and descend from a few wild individuals brought to the DLC in the 1960s. Other non-primate hibernators that were bred in captivity have shown hypothermia and an inability to arouse from torpor, as well as shorter and less deep torpor bouts compared to their wild counterparts^32^. That some individuals lose the capacity to hibernate in captivity may suggest that environmental conditions during ontogenetic development are critical to activate the machinery of hibernation^32^.

**Figure 1:**
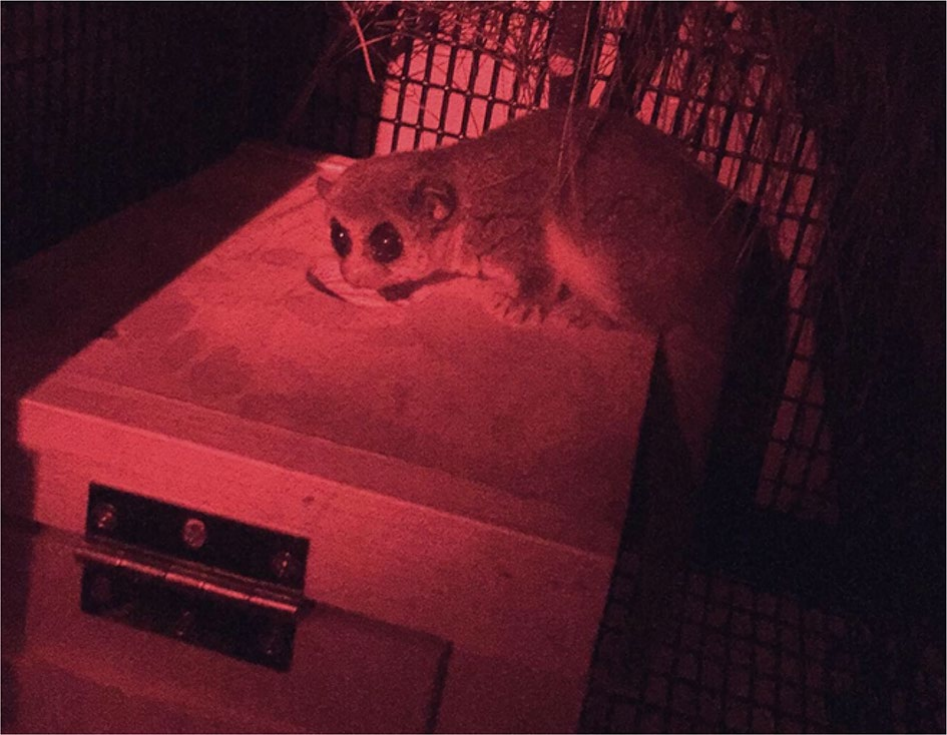
Fat-tailed dwarf lemur “To” about to enter the wooden box used as hibernaculum during the study.

In order to specifically investigate whether fat-tailed dwarf lemurs (*C. medius*) housed at the DLC have the capacity to express hibernation comparable to that of their wild peers^33^, we subjected them to novel husbandry conditions, including lower ambient temperatures and more restricted food availability than was hitherto practiced (Fig. 1). We predict that dwarf lemurs under these conditions will undergo multiday torpor bouts, i.e., hibernate, in order to cope with heightened and sustained energetic demands, and that they will rely on their fat deposits to sustain metabolism during hibernation, as well as arousals from torpor.

## Results

**Figure 2:**
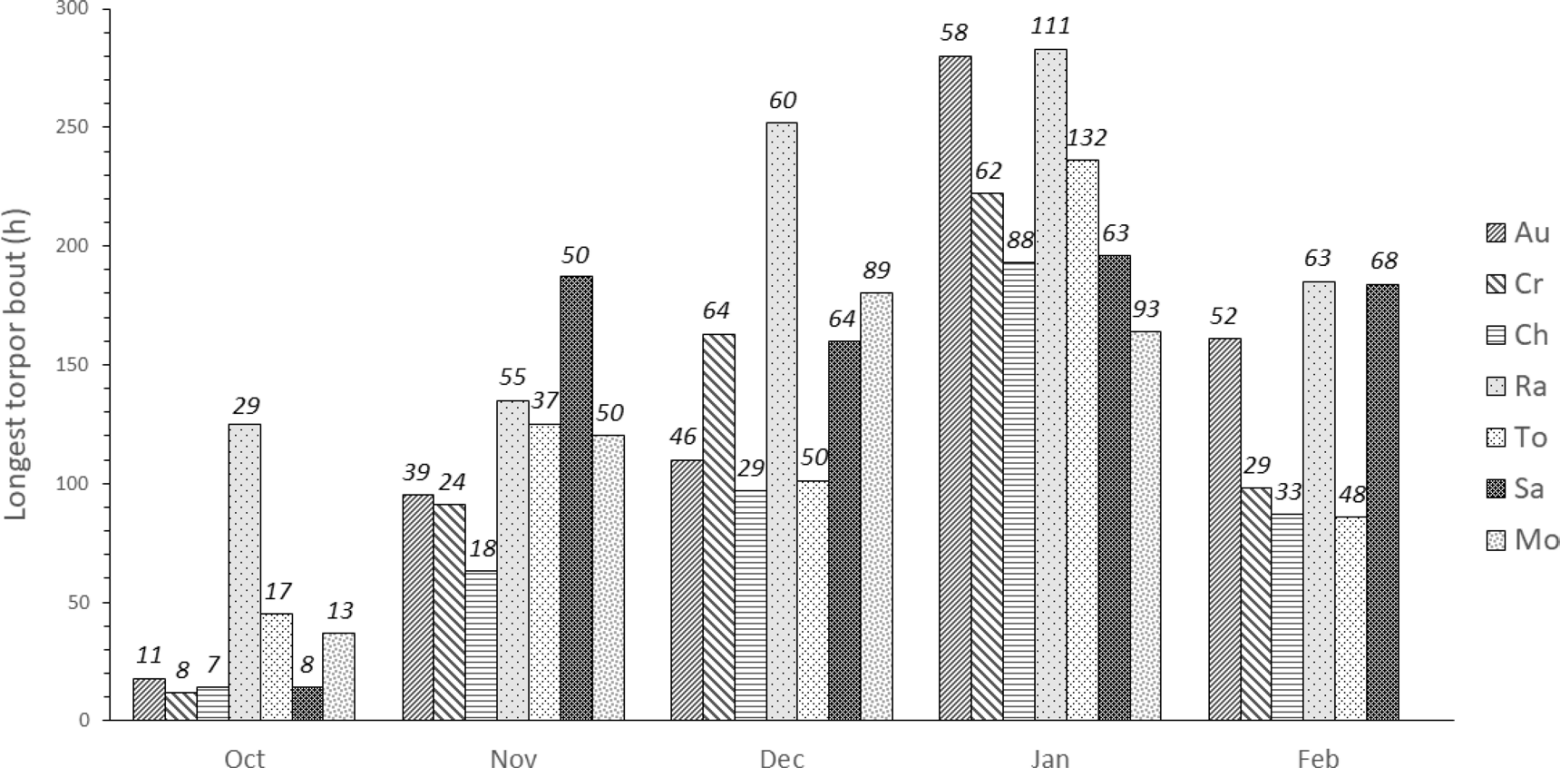
Longest individual torpor bout (bars) and mean torpor bout duration (values above bars) in hours, per individual and month.

We confirm that dwarf lemurs at the DLC can hibernate and express both short torpor bouts (STB, less than 24 h) and long torpor bouts (LTB, more than 24 h). Indeed, dwarf lemurs were in torpor ~ 70% of the time between October 15 and February 10. STB trended towards being more frequent in males than in females (Wilcoxon Rank Test, *p* = 0.08). When compared by month, the number of STB was significantly greater in October and November, compared to later months (Steel–Dwass test, Oct–Jan *p* = 0.0320; Oct–Feb *p* = 0.0271; Nov–Feb *p* = 0.0398). The number of LTB was greater in November and December compared to other months (Steel–Dwaas test, Oct–Nov *p* = 0.0188; Oct–Dec *p* = 0.0144; Nov–Feb *p* = 0.0287; Dec–Jan *p* = 0.0263; Dec–Feb *p* = 0.0215). The longest individual torpor bouts were observed in January, with the exception of individual “Mo” who was removed from the study before the end of the month. (Fig. 2).

**Figure 3:**
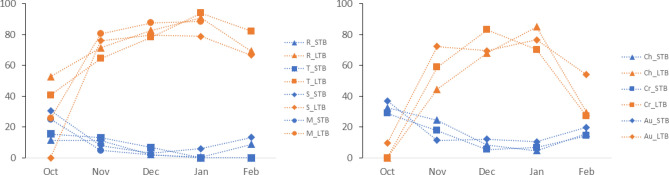
Hours spent in STB (in blue) vs. LTB (orange) per month, females on the left, males on the right, expressed in percentages.

When examining the number of hours spent in STB vs. LTB, dwarf lemurs spent more time expressing STB in October (Steel–Dwass test, Oct–Dec *p* = 0.0273; Oct–Jan *p* = 0.0182) whereas lemurs spent more time expressing LTB in December and January (Steel–Dwaas test, Oct–Nov *p* = 0.0262; Oct–Dec *p* = 0.0177; Oct–Jan *p* = 0.0177) (Fig. 3).

**Figure 4:**
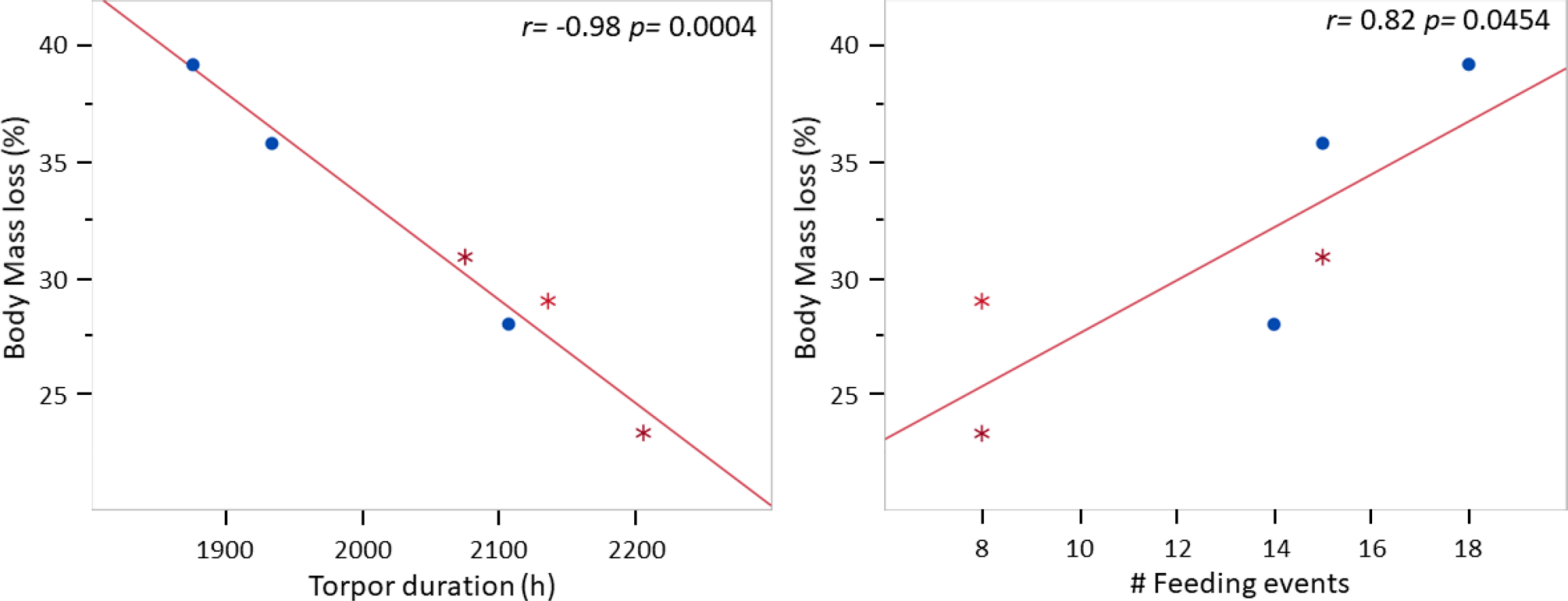
Linear regressions of body mass loss against cumulative torpor occurrence and feeding events. Males are represented by circles, females by asterisks. Pearson correlation coefficients are shown on right upper corner. Data collected between October 15 and January 29.

Based on the numbers of hours in “euthermic” vs. “torpor” states, males were offered food more often than were females, and they generally ate more times than did females, although these differences were not statistically significant, likely due to the small sample size (Table 1). Moreover, body mass loss was higher in males than in females, between 28 and 35% in males, compared to 22 to 28% in females between October 15 and February 7. Tail base circumference (a proxy for fat stores) was also reduced in males to a greater degree (29 to 38%) compared to females (15 to 27%) (Wilcoxon, *p* = 0.08, Table 2). There was a significant negative correlation between body mass loss and torpor duration, as well as a positive significant correlation between body mass loss and feeding events over the course of the study (Fig. 4).

**Table 1:** Number of times food was offered (# Food offerings) compared to how often it was eaten (% Times eaten), body mass loss (% BM loss) and tail base circumference loss (% TBC loss) during the study period.

ID	Sex	# Food offerings	% Times eaten	% BM loss	%TBC loss
"To"	F	26	35	28.4	25.0
"Mo"*	F	20	45	27.1	35.3
"Sa"	F	27	63	26.8	27.4
"Ra"	F	26	42	22.0	15.4
"Ch"	M	38	71	35.0	38.7
"Cr"	M	35	66	29.6	31.6
"Au"	M	26	62	28.4	29.2

*"Mo" individual was removed from study early, data not included in statistical analysis.

**Table 2:** Generalized linear mixed model on the effects of ambient temperature (Twall), night length (Night L), food intake and sex on torpor bout duration.

Variable	Trend	Z value	Pr ( >|z|)
Twall	Longer torpor bouts at lower vs. higher Twall	− 7.30	< 0.0001
Night_L (Dec)	Longer torpor bouts during longer vs. shorter nights	4.39	< 0.0001
Food eaten	Longer torpor when food not eaten vs. eaten	− 4.98	< 0.0001
Sex	Longer torpor bouts by females vs. males	− 2.29	0.022

When we investigated the effect of ambient temperature (Twall), night length (Night_L), food intake (before a torpor bout), and sex on the duration of torpor bouts, we found all variables had a significant effect. Twall was the stronger predictor of torpor duration, with longer bouts occurring at lower temperatures. Night length and food eaten had a similar effect, and sex was the weakest predictor (Table 2).

**Figure 5:**
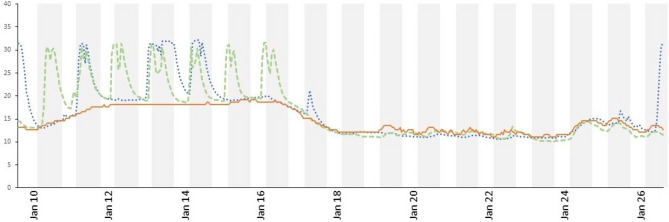
Tsk from female “Ra” (blue, dotted line) and male “Au” (green, dashed line) and Twall (orange, solid line) profiles from Jan 10 to 26 during a “warming” period followed by cold spell. Gray areas show “night” phase.

Although ambient temperature had a significant effect on torpor bout duration, there was also individual variation in the expression of torpor under the same temperature conditions. For instance, during a remarkable warm spell in January, the room temperatures increased by ~ 4 °C and remained at this value (~ 18 °C) for about 6 days, followed by a cooling period. During this time, some dwarf lemurs experienced daily torpor (“Au”) whereas others displayed prolonged torpor (“Ra”). Arousal periods, for both individuals, began around the time of light-switch off, corresponding to the “night” phase. For individual “Au,” hours between arousal periods were: 18, 25, 24, 24, 24, 23, and for “Ra”: 44, 27, displaying cyclicity consistent with circadian regulation. All dwarf lemurs, however, experienced a long torpor bout after cooling of the rooms (Fig. 5).

## Discussion

By exposing members of the DLC dwarf lemur colony to low ambient temperatures and a flexible food-restriction regimen, we demonstrate, for the first time, that captive dwarf lemurs are physiologically capable of sustaining months-long hibernation. As documented for their wild counterparts^24^, longer torpor bouts occurred at lower ambient temperatures, and females showed longer torpor bouts than did males. There was an effect of night length (which increased from October until mid-January) upon torpor bout duration, e.g., with longer torpor bouts in December compared to November, although the rooms were maintained at similar temperatures. This may suggest that regulatory mechanisms other than temperature-dependent circadian clocks may be at play^34^.

The individual and synergistic roles of photoperiod, food, and temperature in modulating the expression of torpor have been previously documented in other small-bodied lemurs, the mouse lemurs^35–37^, which unlike dwarf lemurs, are considered “facultative hibernators”. Although female grey mouse lemurs can experience multi-day torpor bouts in the wild^38^ they have not been observed to do so in captivity. In captive mouse lemurs, torpor expression is generally restricted to short days, although small daily variations in body temperature can be observed during long days^39^. Arousal from torpor in mouse lemurs responds to circadian control entrained by photoperiod^37,40^. Moreover, food restriction is known to facilitate torpor expression, with longer torpor bouts occurring as food restriction is increased^35,36,41^. The combination of food restriction and low ambient temperature shows the greatest effects in mouse lemurs, inducing the longest torpor bouts^37^.

The relationship between torpor and temperature is complex. Mouse and dwarf lemurs can thermocomform to a great range of ambient temperature without “arousing” from torpor. While hibernating, dwarf lemurs can passively track ambient temperature ranging from ~ 10 to 30 °C daily for weeks and months, making arousals unnecessary^42^. Within the temperature range under which dwarf lemurs can express torpor, there appears to be a temperature threshold above which some critical physiological processes can occur. For instance, above 25 °C there is evidence of REM sleep in dwarf lemurs thermoconforming to ambient temperature, but low amplitude brain activity (no sleep) is recorded below 25 °C^29,43^. This temperature baseline may not be exclusive for hibernating lemurs, but for non-primate hibernators as well. Geiser^5^ (p.R190) argues that the “…duration of torpor bouts in hibernators is strongly temperature-dependent and [ ] at high Ta of ~ 20 to 25 °C they often are < 24 h and superficially appear to be daily torpor…”. This may explain why earlier studies on captive dwarf lemurs all failed to report hibernation expression, because the room temperatures were maintained constant between 20 and 25 °C. This may also explain why individual “Au” showed torpor expression comparable to daily torpor during the “warm” week in January.

Unlike wild dwarf lemurs, DLC dwarf lemurs were offered some food during the study and opportunistically ate during the hibernation season. We cannot be certain as to why the dwarf lemurs continued to eat during the study, or whether they, like non-primate hibernators, undergo a remodeling of the intestinal microbiota composition during hibernation^44^. Alternatively, opportunistic feeding may have allowed individuals to limit torpor expression by acquiring energy from the environment. That males fed more times, expressed short torpor bouts more often, and stayed in torpor fewer hours overall than did females (though our sample size is too small to be conclusive) suggest that feeding may have affected torpor dynamics to a degree. Feeding may have increased the frequency of arousals in males which, in turn, increased their energetic costs resulting in greater body mass loss. The more “active” profile of males is also consistent with the torpor optimization hypothesis: torpor expression should be minimized when there is an energy surplus, because of the potential physiological costs associated with metabolic depression^45^. Dwarf lemur females, on the other hand, displayed overall longer torpor bouts than did males, and lost less of their fat reserves by the end of the study. These results are predicted by a modified version of the optimization hypothesis, the thrifty-female hypothesis, that accounts for female reproductive burden: females benefit from maintaining body fat deposits after emergence from hibernation to sustain the costs of imminent reproduction^46,47^.

Although it is tempting to succumb to the idea that torpor expression should be minimized to avoid the associated physiological costs, a question remains as to whether long-term exposure to energy surplus may result in the development of metabolic disorders in organisms that evolved mechanisms to sustain hibernation in the first place^48^. Notably, dwarf lemurs are obligate hibernators in their natural habitats, and they evolved to experience drastic physiological states, alternating between periods of significant fattening in preparation for hibernation, and depletion of fat stores during the hibernation season. These physiological states likely evolved as a mechanism to cope with the environmental hypervariability that characterizes Madagascar, where yearly dry seasons and stochastic weather events limit the availability and stability of high-quality foods^49^. For dwarf lemurs, the reproductive season is thus limited to 4–5 months a year, with females having a short gestation period of ~ 2 months. Young dwarf lemurs display relatively fast growth rates during the first 2 months of life, to achieve ecological independence, and begin depositing fat before they attain adult size. Dwarf lemurs do not achieve sexual maturity until they are ~ 2 or 3 years old^50^. Under captive conditions of constant warm temperature and unrestricted food regimes, however, dwarf lemurs achieve adult size and reproductive maturity within a year and generally experience daily torpor for ~ 3 months^51,52^.

Modulating hibernation expression in dwarf lemurs at the DLC has critical implications for husbandry, as well as research. Although it is difficult to attribute detrimental health effects in the DLC colony to the lack of torpor expression, non-hibernating captive dwarf lemurs do show irregular patterns of body mass gains and losses that do not necessarily correspond to the expected periods of fattening and fasting. At the extreme, some dwarf lemurs maintain consistent weight and significant fat reserves year-round. One can assume that development of diabetes and cataracts that are present in some aged dwarf lemurs at the DLC may be, in part, the result of long accumulation of physiological mismatches under captive conditions. It could be further speculated that the presence of ectopic excess fat retained by dwarf lemurs during the active season may carry long-term physiological costs, as it is associated with the development of metabolic syndrome in non-hibernators^53^.

Thus, exposure to contrasting environmental conditions, leading to the expression of seasonal phenotypes may, in fact, help maintain a healthy dwarf lemur population in captivity, if hibernation is expressed in moderation^54^. A “renaturalized” captive dwarf lemur population will set the stage for future investigations of the mechanisms underlying hibernation expression and its regulation, for example exploring protective mechanisms that allow dwarf lemurs to tolerate spikes in sugar or lipid metabolism without showing signs of illness^20^. This is particularly promising as dwarf lemurs, the closest relatives to humans that hibernate, are emerging as excellent animal models for understanding the mechanisms of metabolic extremes and their implications for ageing and metabolic disorders, such as diabetes and metabolic syndrome, that are known all too frequently in humans.

## Methods

### Housing conditions

**Figure 6:**
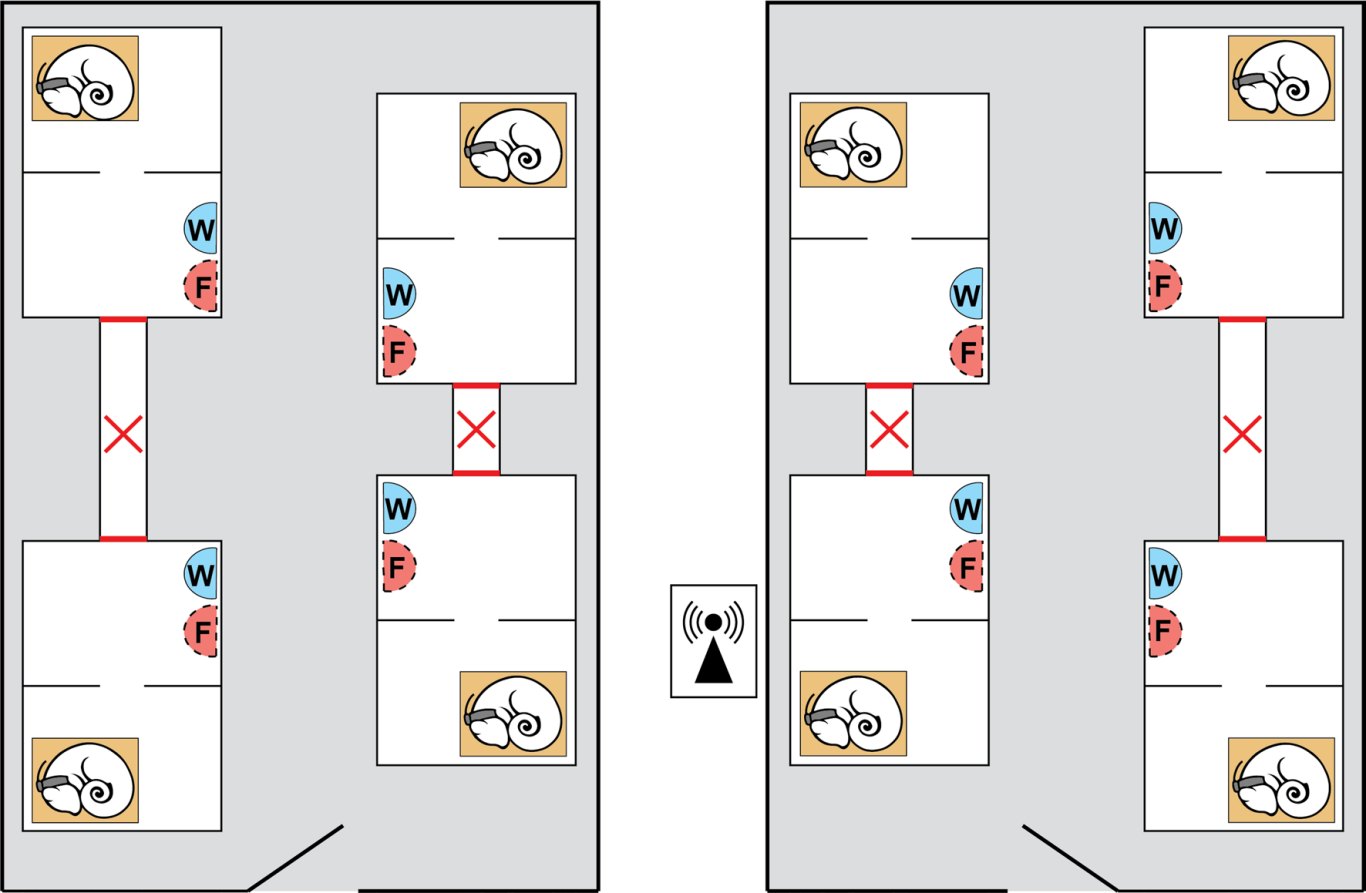
Schematics of the experimental setting. Grey boxes represent the rooms; white boxes represent the individual lemurs’ enclosures; brown boxes represent the wooden nest/”hibernacula”; F and W reflect the placement of food and water dishes, respectively.

The dwarf lemurs were housed in two separate temperature-controlled rooms, four individuals per room between September 30 (Room A) or October 1 (Room B) 2019 to February 14, 2020 (Fig. 6). Each dwarf lemur was kept in a separate enclosure, divided in quarters with open access between quarters. The dimensions were 24″ L × 20″ W × 36″ H per quarter. Doors that allowed social contact between individuals were shut, but the dwarf lemurs were able to see and smell roommates. Each enclosure was furnished with a single wooden box (10″ L × 5.5″ W × 7″ H), to function as a hibernaculum. The box had a small hole on top, simulating a tree hole entrance, and a small door on the front, opened by staff for daily checking of the lemurs inside the boxes. We documented the timing of box openings to check whether daily checks correlated with arousal timing. We found no relationship, suggesting dwarf lemurs were not disturbed by this monitoring system.

For acclimatization purposes, when dwarf lemurs were transferred to the rooms, they were maintained at ~ 25 °C for a week (between October 1–7), and at ~ 20 °C the following week (between October 7–14). From October 15 until February 10, the temperature was maintained between 10 and 15 °C. Despite attempts to keep temperatures relatively constant, there were daily fluctuations, sometimes exceeding 4 °C (e.g. January 17th). On February 10, when the study ended, room temperatures were increased to ~ 20 °C to begin acclimatization of the individuals to “summer” temperatures. The temperature of each room (Twall) was recorded hourly by an ibutton attached to a wall, located halfway between the door and the opposite end of the room, and at breast height (the fours wooden boxes were located at either side of the ibutton and at higher or lower positions). Another ibutton was located inside an empty box located inside one of the rooms. When comparing the “inside the box” temperature against “wall” temperature, differences were less than 1 °C and considered negligible. Thus, we used Twall for the purposes of comparisons with skin temperature (Tsk) from radiocollars. Room A was consistently colder than Room B during the study period by ~ 1 °C (Table 3). Water was available ad libitum and changed every day. Food was offered once an individual dwarf lemur had accrued 24 h of activity, per protocols (see below).

**Table 3:** Temperature values (°C) from ibuttons placed on the wall of temperature-controlled rooms during the study period, expressed as monthly absolute minimum, absolute maximum and means.

Month	Room A			Room B		
	Min	Max	Mean	Min	Max	Mean
October^a^	12.6	20.8	15.6	13.9	22.1	16.9
November	10.8	17.2	12.8	11.4	18.4	13.8
December	11.1	17.1	12.6	11.1	18.1	13.4
January	11.1	17.6	12.8	11.1	19.1	13.7
February	11.1	19.6	13.6	11.1	19.6	14.1

^a^Including data from October 15 to 31.

### Photoperiod

We employed a shifting photoperiod, from “winter”-like days (shortest 9:30 h light) to “summer”-like days (longest 14:30 h light). Daylight increased from January to June and decreased from July to December, following a northern hemisphere schedule, i.e., opposite to Madagascar. Daylight changes were programmed to occur in 30 min periods every other week. At the time of transfer to the temperature-controlled rooms, on September 30 and October 1, the dwarf lemurs experienced shortening photoperiods until January 13, when daylight began to increase (Table 4).

**Table 4:** Changes in daylength at the DLC to approximate North Carolina, USA photoperiod.

Date	Light phase (h)	Dark phase (h)
Sept. 21	12:00	12:00
Oct. 6	11:30	12:30
Oct. 21	11:00	13:00
Nov. 2	10:30	13:30
Nov. 17	10:00	14:00
Dec. 2	9:30	14:30
Jan. 13	9:50	14:10
Jan. 28	10:00	14:00
Feb. 12	10:30	13:30

### Study subjects, telemetry, skin temperature measurements

We selected eight adult dwarf lemurs, four females and four males aged between 6 and 15 years, from the DLC colony to participate in the torpor study (Table 5). Two dwarf lemurs were removed early from the study: male “Os” was removed on December 14 when reaching his critical low value for body mass (140 g) and when showing difficulties arousing from torpor. Inability to arouse from torpor and the failure to adopt a curled-up position have been observed in other captive bred hibernators^32^; female “Mo” was removed from the study on January 16 after achieving her critical low value for body mass (142 g). We kept data from “Mo” in the analysis, however, because her behavior had been considered normal until her removal. Critical values for body mass were calculated, on an individual basis, as the lowest weight ever recorded per lemur in adulthood minus 10%. These values ranged between 140 and 158 g.

**Table 5:** The dwarf lemurs selected for the torpor study and their body masses (BM) and tail base circumferences (TBC) at the onset and end of the study.

ID	Sex	Entry date	Entry BM (g)	Entry TBC (cm)	Exit date	Exit BM (g)	Exit TBC (cm)
“Ch”	Male	9/30	302	7.5	2/7	171	4.6
“To”	Female	9/30	273	7.2	2/7	174	5.4
“Mo”	Female	9/30	236	7.0	1/13	150	4.4
“Os”	Male	9/30	243	7.2	Removed early from study		
“Au”	Male	10/1	253	7.3	2/7	166	5.1
“Ra”	Female	10/1	269	7.8	2/7	195	6.6
“Cr”	Male	10/1	273	7.9	2/7	188	5.3
“Sa”	Female	10/1	270	8.3	2/7	180	6.1

At the beginning of the study and every ~ 15 days, the dwarf lemurs were weighed and their tail base circumference (a proxy for fat reserves) was measured using a measuring tape. Because handling individuals and removing them from nest boxes generally triggered an arousal, we tried to time weighing sessions with “natural” arousals, which we could detect by checking dwarf lemurs’ Tsk values displayed on the receiver. Due to Institutional regulations, however, we could only delay weighing session up to 5 days from the scheduled date. We documented whether individuals were “in torpor” or “euthermic” at the time of weighing. Data included in the analysis were collected between October 15, 2019 and February 10, 2020. This corresponds to the time periods when the temperature of the rooms was maintained between ~ 10 and 15 °C.

The dwarf lemurs were fitted with small external radio collars with variable pulse rate transmitters (M1550, ~ 3.5 g, Advanced Telemetry Systems, Isanti, MN; collar size/body mass ratio of < 4%). Pulse rates varied according to temperature of the external sensor, which was in contact with the dwarf lemurs’ skin around the neck (Tsk). Those pulse rates were automatically converted to temperature values in degrees Celsius by a receiver with datalogger (R4500, Advanced Telemetry Systems, Isanti, MN). The receiver, which was placed outside of the rooms, scanned transmitters continuously and stored the transmitters’ temperatures hourly. Radio collars were fitted to be gently pressed against the dwarf lemurs’ necks to provide skin temperature estimates. The tighter the contact (e.g., when an individual is curled-up) the better the approximation of body temperature. Although Tsk is only a proxy for body temperature, we could clearly differentiate “euthermic” vs. “torpor” states. Originally, we set 29 °C or less as the temperature threshold for torpor. After visual inspection of the data and observations of dwarf lemurs during daily checks, we determined that 22.5 °C or less was a more conservative value, so as not to overestimate torpor expression. For instance, individuals were seen outside of the wood box, moving around at Tsk 24 °C. This relatively low temperature value for activity may be explained by transmitter’s sensor picking up dwarf lemur’s skin temperature (presumably higher at the time of moving around), but also ambient temperature (between 10 and 15 °C).

Every morning at ~ 8:00, the authors downloaded Tsk data from the previous 24 h for all dwarf lemurs to a laptop computer. Hours in “torpor” (< 22.5 °C) vs. “euthermic” (≥ 22.5 °C) were counted and reported on the individuals’ data sheets. If the sum of the “euthermic” hours since the last feeding event reached 24 h, food was prepared and placed inside the individual’s enclosure along with daily water replacement.

### Food restriction

For the first 2 weeks of the study (October 1 and 15), the dwarf lemurs were offered a standard “winter” daily diet, consisting of fruit/veggie mix and Old-World monkey chow per approved standard DLC husbandry protocols (caloric content ~ 15 kcal vs. standard “summer” diet caloric content ~ 40 kcal). Once the rooms were cooled down to ~ 15 °C, we put in place a food restriction protocol: the dwarf lemurs were offered the standard “winter” diet if they accumulated a minimum of 24 h of activity since the last time food was offered. Each morning, food bowls were removed and inspected for leftovers. Scores were noted on each individual’s datasheet as follows: 3 = all food left, 2 = half food left, 1 = little food left, 0 = nothing left. For the purpose of the analysis, we lumped scores 1–3 as “food eaten” and score 0 as “food uneaten”.

All research and experimental protocols used in this study were approved by the Duke University Institutional Animal Care and Use Committee, under protocol A263-17-12, and the Duke Lemur Center Research Committee (MO-11-18-6). These protocols followed guidelines established by the Guide for the Care and Use of Laboratory Animals of the National Institutes of Health.

### Statistical analysis

Torpor bouts were categorized as “short torpor bouts” (STB) if they lasted 24 h or less, and “long torpor bouts” (LTB) if they were longer than 24 h. We also calculated average Tsk and average Twall during torpor bouts and recorded whether torpor bouts started soon after food had been ingested (within 24 h of “food intake”, scores 1–3). We ran non-parametric tests, Wilcoxon and Steel–Dwass, to compare body mass and tail base circumference between sexes, and torpor bout frequency across months (October to February), respectively, and we calculated Pearson correlation coefficients between body mass loss (in %) and torpor duration (in hours) or feeding events in JMP (version Pro 15.0.0^55^). We ran a linear mixed model using the glmmADMB package (version 0.8.3.3^56^) and R software program (version 3.3.3^57^) in Rstudio (version 1.1.463^58^), using log-transformed torpor bouts (in hours) as response variable (Suppl. Mat). We used Twall (continuous variable in degrees C), night length (continuous variable in hours), food eaten (categorical variable: yes or no), and sex (categorical variable: male or female) as explanatory variables and individual lemur as a random variable. We determined that the log-transformed gaussian binomial distribution produced the best fit model for our data (AIC: 1087).

## Supplementary Information

Supplementary Information.
